# Fique Fabric: A Promising Reinforcement for Polymer Composites

**DOI:** 10.3390/polym10030246

**Published:** 2018-02-28

**Authors:** Sergio Neves Monteiro, Foluke Salgado de Assis, Carlos Luiz Ferreira, Noan Tonini Simonassi, Ricardo Pondé Weber, Michelle Souza Oliveira, Henry A. Colorado, Artur Camposo Pereira

**Affiliations:** 1 Materials Science Program, Military Institute of Engineering, IME, Praça General Tibúrcio 80, Urca, Rio de Janeiro 22290-270, Brazil; snevesmonteiro@gmail.com (S.N.M.); foluke.assis@hotmail.com (F.S.d.A.); cferreira@ime.eb.br (C.L.F.); noantoninisimonassi@gmail.com (N.T.S.); rpweber@ime.eb.br (R.P.W.); oliveirasmichelle@gmail.com (M.S.O.); 2 CCComposites Laboratory, Universidad de Antioquia, UDeA, Calle 70, No. 52-21, Medellin 050010, Colombia; henry.colorado@udea.edu.co

**Keywords:** fique fabric, composites, polyester matrix, thermal dynamic mechanical behavior, ballistic performance

## Abstract

A relatively unknown natural fiber extracted from the leaves of the fique plant, native of the South American Andes, has recently shown potential as reinforcement of polymer composites for engineering applications. Preliminary investigations indicated a promising substitute for synthetic fibers, competing with other well-known natural fibers. The fabric made from fique fibers have not yet been investigated as possible composite reinforcement. Therefore, in the present work a more thorough characterization of fique fabric as a reinforcement of composites with a polyester matrix was performed. Thermal mechanical properties of fique fabric composites were determined by dynamic mechanical analysis (DMA). The ballistic performance of plain woven fique fabric-reinforced polyester matrix composites was investigated as a second layer in a multilayered armor system (MAS). The results revealed a sensible improvement in thermal dynamic mechanical behavior. Both viscoelastic stiffness and glass transition temperature were increased with the amount of incorporated fique fabric. In terms of ballistic results, the fique fabric composites present a performance similar to that of the much stronger Kevlar^TM^ as an MAS second layer with the same thickness. A cost analysis indicated that armor vests with fique fabric composites as an MAS second layer would be 13 times less expensive than a similar creation made with Kevlar™.

## 1. Introduction

**Figure 1:**
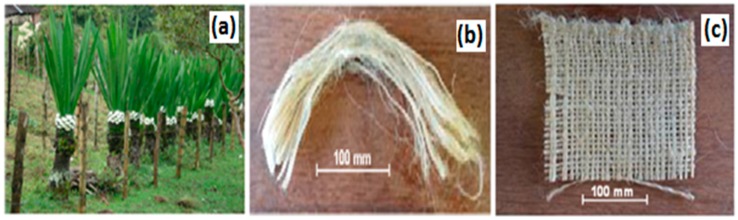
Plantation of fique (*Furcraea andina*) in Colombia (**a**), bundle of fique fibers (**b**), and as-received piece of fique fabric (**c**).

The beginning of this new century witnessed a surge in research works dedicated to the use of fibers extracted from plants in engineering applications. Several specialized and review articles [1,2,3,4,5,6,7,8,9,10,11,12,13,14,15] emphasized the use of these natural lignocellulosic fibers and their fabrics as reinforcements of polymer composites, competing with synthetic fibers. Environmental sustainability goals associated with lower cost, societal benefits, and some technical advantages favor the substitution of natural fiber and fabric for glass fiber [16] and aramid fabric [17]. Industrial components, mainly in automobile fabrication [18,19,20], increasingly employ natural fiber and fabric composites. A specific industrial sector in which fibers and fabrics are of relevance is that of armor vests. Originally, fiber glass was extensively used [21,22] and carbon fiber-reinforced polymer composites were also considered [23]. Today, synthetic aramid fabrics such as Kevlar™ (Du Pont, Richmond, VA, USA) [24] and Twaron™ (Teijin Aramid, Conyers, GA, USA) [25] as well as ultra-high molecular weight polyethylene fibers, such as Spectra™ (Spectra Energy Corporation, Houston, TX, USA) [26] and Dyneema™ (DSM Dyneema LLC, Stanley, NC, USA) [27], are major choices for personal armor vests. Recent publications [28,29,30,31,32,33] revealed that natural fibers as well as corresponding fabrics-reinforced polymer composites display comparable ballistic performance to Kevlar^TM^. In spite of numerous publications dedicated to this subject, the growing interest for engineering applications is continuously demanding research works on less common promising natural fibers and fabrics. An example is the relatively unknown fiber extracted from the leaves of an Andean plant (*Furcraea andina*). This fique fiber has been brought to attention for its potential as a composite reinforcement [34,35,36,37,38,39]. Figure 1 illustrates: (a) fique plantation in Colombia and (b) a bundle of fique fibers extracted from the leaves of the plant. For practical use, the fique fabric is yearlong found in the Colombian market and largely available for common applications, mostly as sackcloth for agricultural products. Owing to its national importance, the federal government of Colombia controls both the price and quality of the fique fiber, which is stored and distributed for industrial processing, including as a textile. The extraction cost, from plantation to clean fibers (see Figure 1a,b), is around US$0.14, while the consumer price varies from US$0.36 to US$0.45. Fiber surface modification [34] and thermal degradation [35] as well as bending [36] and tensile [37,38] properties of fique fiber-reinforced composites were preliminarily investigated. Moreover, an inverse correlation between the density and the diameter of fique fibers was established [39]. As for fique fabric, either woven or non-woven, characterization and properties of polymeric-reinforced composites are still not found in the scientific literature. In particular, thermal dynamic mechanical properties, which are important for engineering applications, were not mentioned for fique fabric in the only review [40] dedicated to polymer composites reinforced with less common natural fiber-based materials. These properties might be obtained by dynamic mechanical analysis (DMA) and could provide viscoelastic behavior as well as temperature effect on distinct dynamic moduli. Any possible engineering application of fique fabric-reinforced composites, particularly in personal armors, will demand additional information on thermal dynamic mechanical and ballistic properties, which is the objective of the present work.

## 2. Materials and Methods

### 2.1. Materials Source and Process

Fique fabric commercially available in Colombia was supplied by Compañia de Empaques, Itagüí, Antioquia, Colombia. A piece of as-received fabric is shown in Figure 1c. An average equivalent diameter of 0.18 mm and an average density of 667 kg/m^3^ for the fique fiber, Figure 1b, were reported elsewhere [39]. The areal density of the plain-woven fabric, Figure 1c, investigated in this work was found to be 0.036 kg/m^2^. An isophtalic polyester resin hardened with 1% methyl-ethyl-ketone, fabricated by Dow Chemical and supplied by Resinpoxy, Rio de Janeiro, Brazil, was used as the composite matrix. Composites were prepared by accommodating the previously dried fabric (60 °C for 24 h) in a steel mold, and then pouring the still fluid resin-hardener mixture between fabric layers. In this way, 120 × 150 × 50 mm^3^ plates of laminate composites with 10, 20, and 30 vol % fique fabric associated with 4, 8, and 12 layers were respectively produced. These plates were kept under 3 MPa pressure for 24 h, at room temperature (25 °C), until complete solid cure. Test samples were cut from the laminate composite plates and standard flexural specimens with 10, 20, and 30 vol % fabric, as well as neat polyester for control, were prepared.

### 2.2. Dynamic Mechanical Analysis (DMA)

DMA tests were performed in fique fabric composites, using flexural specimens according to standard. All analyses were conducted in triplicate to evaluate the degree of dispersion in corresponding curves. Less than 10% difference was found between curves for identical conditions, basically confirming the same results. Therefore, only one typical curve is used to illustrate each condition.

Each DMA test was carried out in the interval from −20 to 160 °C in a model Q/800 TA Instruments (New Castle, DE, USA) in three-point flexural mode, 1 Hz frequency, and a heating rate of 3 K/min (3 °C/min) under nitrogen atmosphere. The storage modulus (E′), loss modulus (E″), and tangent delta curves were provided by the equipment. Prismatic standard specimens with dimensions of 50 × 13 × 3 mm were used for the tests.

### 2.3. Ballistic Tests

**Figure 2:**
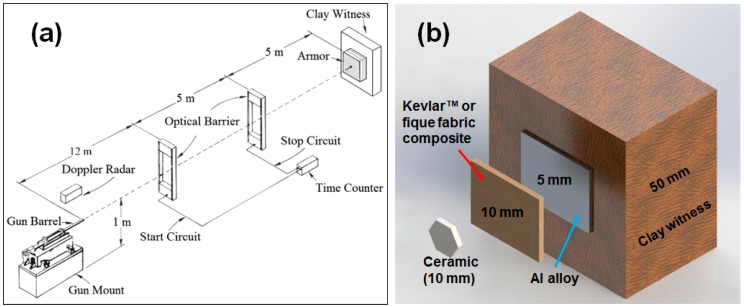
(**a**) Schematic exploded view of the ballistic experimental setup and (**b**) schematic illustration of the investigated multilayered armor system (MAS), as a target placed ahead of a clay witness block.

In addition to DMA, preliminary ballistic tests were carried out according to the NIJ 0101.06 standard using 7.62 × 51 mm^2^ NATO military ammunition with a 9.7 g projectile propelled from a gun barrel. The tests were conducted at the Brazilian Army shooting facility, CAEX, in the Marambaia Peninsula, Rio de Janeiro, with three samples of 10 and 20 vol % of fique fabric for each type of multilayered armor system (MAS) [41,42]. Figure 2a shows the exploded view of the ballistic test setup while Figure 2b illustrates, schematically, the MAS composed of a front ceramic followed by a layer of fique fabric composites and backed by an aluminum alloy sheet. The MAS is set as a target together with a block of so-called clay witness that simulates a human body and should only allow penetration to a certain depth. According to the standard NIJ 0101.06, the measured depth of indentation is limited to 44 mm in order to avoid lethal trauma to the MAS wearer. Measurements were performed with a laser sensor caliper with 0.01 mm of precision.

### 2.4. Fracture Microscopy

The ruptured surface of each MAS component after the ballistic test was analyzed by scanning electron microscopy (SEM) in a model QUANTA FEG250 Fei (Thermo Fisher Scientific, Hillsboro, OR, USA) microscope operating with secondary electrons at 20 kV.

## 3. Results and Discussion

### 3.1. Dynamic Mechanical Analysis (DMA)

**Figure 3:**
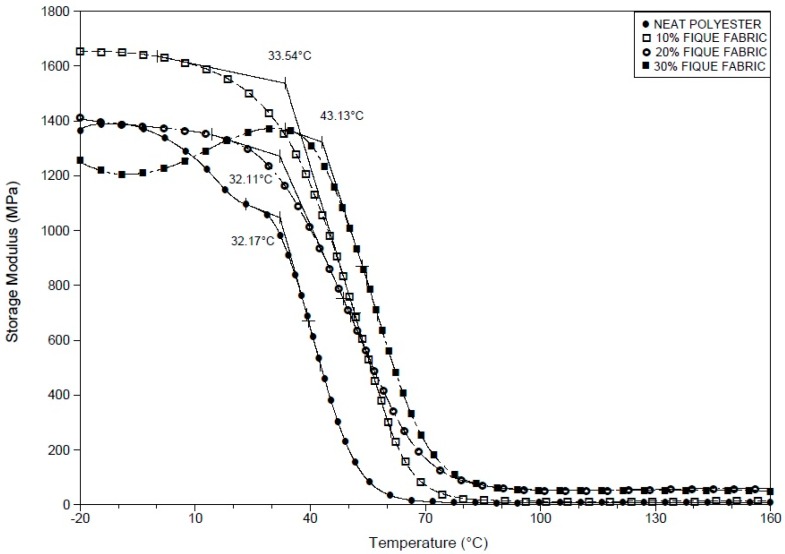
Storage modulus (E′) curves for the neat polyester resin and for the composites reinforced with fique fabric.

**Figure 4:**
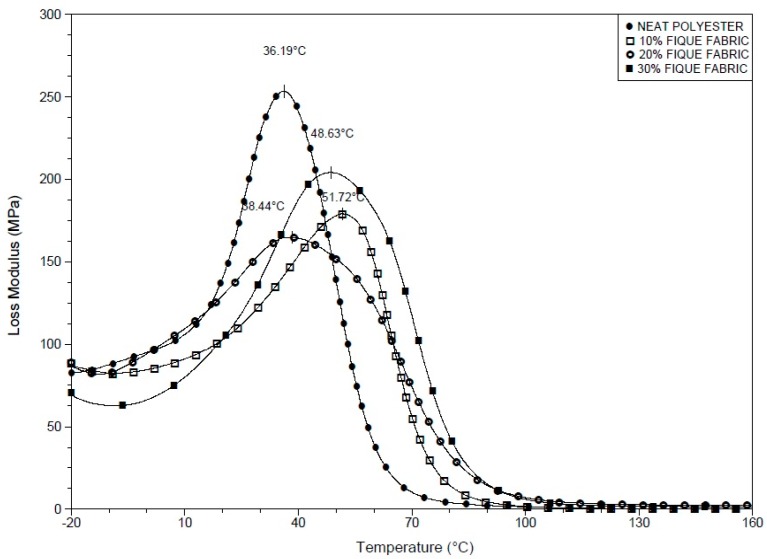
Loss modulus (E″) curves for the neat polyester resin and for the composites reinforced with fique fabric.

**Figure 5:**
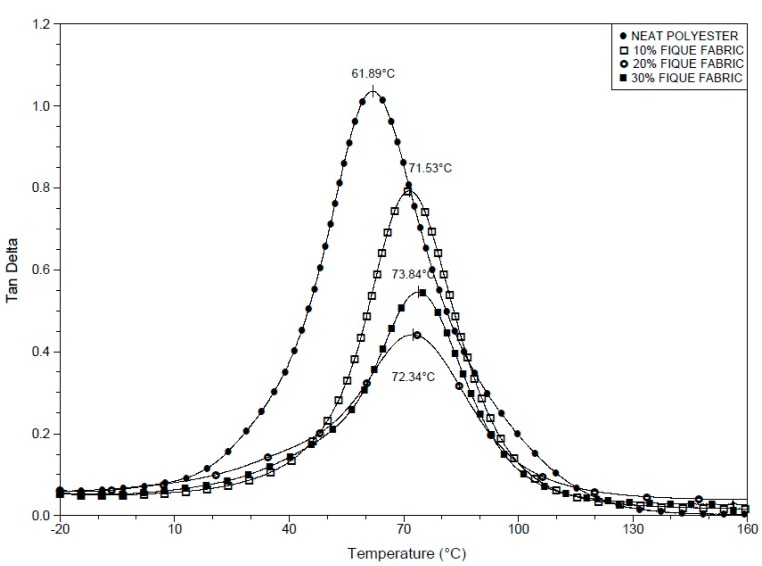
Tan δ curves for the neat polyester resin and for the composites reinforced with fique fabric.

Figure 3, Figure 4 and Figure 5 respectively show the DMA storage modulus (E′), loss modulus (E″), and tangent delta (tan δ) curves for the neat polyester resin and fique fabric-reinforced composites. Figure 3 compares the variation of the storage modulus (E′) with temperature for the neat polyester and different composites investigated. For all temperature levels, from 25 °C up to 70 °C, the E′ values for the fique fabric composites are significantly higher than that of the neat polyester resin. In fact, the value of E′ is directly related to the material’s ability to withstand mechanical loads with recoverable viscoelastic deformation. Consequently, the results in Figure 3 above room temperature indicate a substantial raise in the viscoelastic stiffness of the polyester with increasing incorporation of fique fabric, without loss in recoverable deformation. Moreover, while an accentuated decrease in stiffness begins to occur around 10 °C for the neat polyester, the 30 vol % fique fabric composite remains stiffer up to 40 °C. As the temperature increases, there is a rapid decrease in the E′ value from about 40 °C until a plateau of less than 100 MPa is reached.

Figure 4 compares the variation of the loss modulus (E″) with temperature for the neat polyester resin and the investigated composites. All of the curves of this figure go through a well-defined maximum value that can be associated with the relaxation peak α [43]. This relaxation is attributed to the mobility of the chains in the crystalline phase of the polymer, which in this work is the polyester matrix [44]. For the fique fabric composites, the peaks α in the E″ curves are shifted by about 15 °C for higher temperatures. Kalusuraman et al. [43] suggested that the α peaks in E″ could be related to the onset of the glass transition temperature (*T*_g_) of the polymeric matrix. Therefore, the results in Figure 4 revealed that the incorporation of fique fabric restrains the mobility of the polyester chains and retards the formation of an amorphous structure. This mechanism also explains the relative shift to higher temperature (10 to 40 °C) of the fique fiber composites viscoelastic softening, Figure 3, as compared to the neat polyester.

The variation of the tan δ with the temperature for the neat polyester resin and the composites incorporated with fique fabric is shown in Figure 5. It can be seen in this figure that the composites exhibit peaks with lower amplitude and shifted to relatively higher temperatures as compared to the neat polyester resin. This suggests, as also observed for the storage modulus in Figure 3 and loss modulus in Figure 4, that the fique fabric effectively interacts with the polyester matrix chains, impairing their mobility and reducing their structural damping ability. The tan δ peaks are attributed to the upper limit of *T*_g_ [43]. Moreover, the decrease in peak amplitude and increase in temperature, Figure 5, also suggest a higher attenuation in internal vibration and a shift to higher temperature of *T*_g_ with the increasing amount of fique fabric in the polyester matrix. Consequently, the results in Figure 5 indicate that the incorporation of fique fabric not only reduces the mobility of the polyester chains, but also prevents the disruption of their structural organization. Consequently, the end of the polyester matrix transition from the glass to the rubbery state could occur at higher temperatures.

### 3.2. Ballistic Tests

Table 1 presents the average depth measured in the clay witness for the different MAS targets investigated. For application in armor vests, the fique fabric composite is lighter and significantly cheaper than the Kevlar™. These are factors that are further discussed and might play practical advantages in considering the substitution of fique fabric composites for Kevlar™ in an MAS for personal protection against high velocity projectiles, such as the 7.62 × 51 mm projectile.

**Figure 6:**
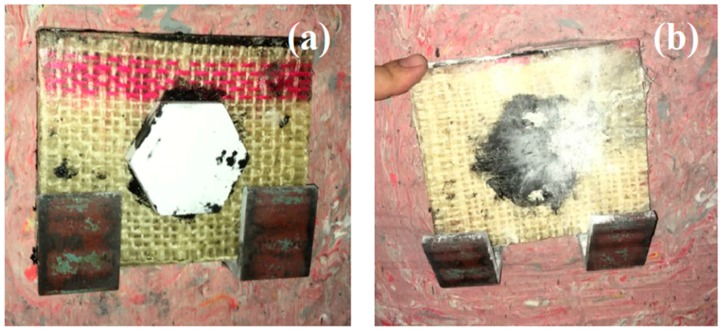
Typical aspect before (**a**) and after (**b**) ballistic test of MAS targets with a second layer of fique fabric-reinforced polyester composite.

Figure 6a illustrates the aspect of the different MAS targets after the ballistic tests. In Figure 6b, it can be seen that the ceramic has disappeared by complete shattering.

**Figure 7:**
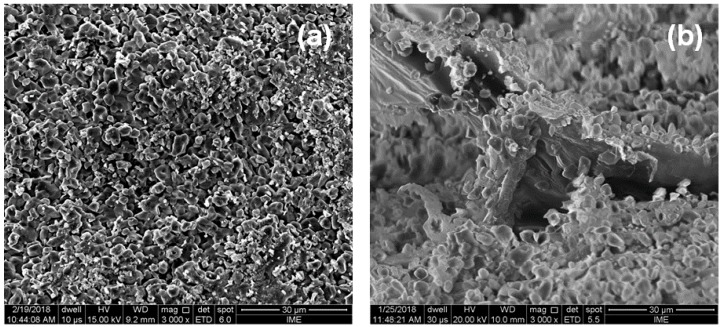
(**a**) Fracture surface of a particle from the ceramic after the ballistic test and (**b**) fracture surface of 20 vol % fique fabric composite covered with ceramic fragments.

After ballistic tests, the fracture surface of MAS components were observed by SEM. Figure 7a shows the expected inter-crystalline brittle fracture surface of a collected macroscopic ceramic particle, which is almost splitting into microscopic fragments associated with grains. As indicated by Medvedovski [46], a 7.62 mm projectile causes different kinds of cracks to be formed during the impact. Figure 7b shows the ability of the 20 vol % fique fabric composite second layer, in an MAS with a ceramic front, to collect fragments generated from the ballistic impact shown in Figure 7a. This ability does not require stronger fibers but mechanisms of mechanical incrustation as well as fragment attraction by Van der Waals forces and static charges on the fiber surface, of either synthetic Kevlar™ [47] or natural fiber-based composites [28,29,30,31,32,33].

### 3.3. Cost Comparison

A cost model associated with the use of fique fabric composites in comparison to conventional engineering composites is presented in Table 2. The basic costs were obtained from the literature for polyester, epoxy, aramid laminates (Kevlar^TM^), and glass fiber [48], as well as for fique fiber/fabric [49] and for sisal, jute, curaua, and piassava [50].

In this table, is it shown that Kevlar^TM^ (aramid fiber laminate) is the most expensive composite, more than 13 times the price of any common natural fiber composite, including the presently investigated fique composites. Even a less expensive glass fiber composite is more than five times as costly as any natural fiber composite. Therefore, the incorporation of any common natural fiber-based material, including fique fabric, reduces the price of the composite.

## 4. Summary and Conclusions

- The introduction of fique fabric raises the viscoelastic stiffness level and tends to shift the curves of the storage modulus (E′) to higher temperatures. This leads to a delay in the onset of the thermal softening of the composite. The peak α of the loss modulus (E″) is also shifted to higher glass transition temperatures (*T*_g_), indicating less mobility in the polyester resin chains of the matrix by interaction with the fique fabric. The maximum in tan δ curves, associated with end of *T*_g_, suffers not only a reduction in its amplitude but also a shift towards higher temperatures with the introduction of fique fabric. Hence, a high attenuation of internal vibration and increase in *T*_g_ occur with an increasing amount of fique fabric in the polyester matrix.
- A multilayered armor system (MAS), in which conventional Kevlar™ was replaced by a polyester matrix composite reinforced with 10 or 20 vol % of fique fabric as second layers, attended the NIJ trauma limit after ballistic tests with 7.62 mm ammunition. The depth of penetration into 20 vol % fique fabric composite, 15 mm, demonstrated this composite to be more efficient than conventional Kevlar™ with 23 mm as a second MAS layer.
- More than ballistic performance, the significantly lower cost in association with the environmental and societal benefits of using a natural material favor the substitution of fique fabric composite as an MAS second layer. As an economical advantage, armor vests with fique fabric composites would be 13 times cheaper than similar ones made with Kevlar™.

## Figures and Tables

**Table 1:** Average depth of penetration in the clay witness backing different MAS of fique fabric composites.

Intermediate Layer Material	Depth of Penetration (mm)
10 vol % fique fabric	17 ± 3
20 vol % fique fabric	15 ± 3
Kevlar™	23 ± 3 [45]

**Table 2:** Cost model for different fabric composites.

Composite Material	Cost (US$/Kg)	Reference
64.8 vol % aramid laminate/epoxy	49.59	[48]
72 vol % glass fiber/epoxy	18.06	[48]
30 vol % sisal fiber/polyester	3.23	[49]
30 vol % jute fiber/polyester	3.24	[50]
30 vol % curaua fiber/polyester	3.19	[50]
30 vol % piassava fiber/polyester	3.21	[50]
20 vol % fique fabric/polyester	3.61	Present Work
30 vol % fique fabric/polyester	3.26	Present Work
